# Default Mode and Executive Networks Areas: Association with the Serial Order in Divergent Thinking

**DOI:** 10.1371/journal.pone.0162234

**Published:** 2016-09-14

**Authors:** Jarmo Heinonen, Jussi Numminen, Yevhen Hlushchuk, Henrik Antell, Vesa Taatila, Jyrki Suomala

**Affiliations:** 1 NeuroLab, Laurea University of Applied Sciences, Espoo, Finland; 2 Aalto NeuroImaging, Aalto University, AALTO, Espoo, Finland; 3 Helsinki Medical Imaging Center, Töölö Hospital, University of Helsinki, Helsinki, Finland; 4 Neurosurgery Research Group, Biomedicum Helsinki, Helsinki, Finland; 5 Turku University of Applied Sciences, Turku, Finland; 6 Department of Psychology and Brain Sciences, University of California Santa Barbara, Santa Barbara, California, United States of America; University of Cambridge, UNITED KINGDOM

## Abstract

Scientific findings have suggested a two-fold structure of the cognitive process. By using the heuristic thinking mode, people automatically process information that tends to be invariant across days, whereas by using the explicit thinking mode people explicitly process information that tends to be variant compared to typical previously learned information patterns. Previous studies on creativity found an association between creativity and the brain regions in the prefrontal cortex, the anterior cingulate cortex, the default mode network and the executive network. However, which neural networks contribute to the explicit mode of thinking during idea generation remains an open question. We employed an fMRI paradigm to examine which brain regions were activated when participants (n = 16) mentally generated alternative uses for everyday objects. Most previous creativity studies required participants to verbalize responses during idea generation, whereas in this study participants produced mental alternatives without verbalizing. This study found activation in the left anterior insula when contrasting idea generation and object identification. This finding suggests that the insula (part of the brain’s salience network) plays a role in facilitating both the central executive and default mode networks to activate idea generation. We also investigated closely the effect of the serial order of idea being generated on brain responses: The amplitude of fMRI responses correlated positively with the serial order of idea being generated in the anterior cingulate cortex, which is part of the central executive network. Positive correlation with the serial order was also observed in the regions typically assigned to the default mode network: the precuneus/cuneus, inferior parietal lobule and posterior cingulate cortex. These networks support the explicit mode of thinking and help the individual to convert conventional mental models to new ones. The serial order correlated negatively with the BOLD responses in the posterior presupplementary motor area, left premotor cortex, right cerebellum and left inferior frontal gyrus. This finding might imply that idea generation without a verbal processing demand reflecting lack of need for new object identification in idea generation events. The results of the study are consistent with recent creativity studies, which emphasize that the creativity process involves working memory capacity to spontaneously shift between different kinds of thinking modes according to the context.

## Introduction

Scientific findings [1–3] have suggested a two-fold structure of the cognitive processes that includes heuristic and explicit components. By using the heuristic thinking mode, people automatically process information that tends to be invariant across days [2]. This mode allows an individual to behave according to a conventional habit, which includes the typical practical and mental actions of everyday work [4]. In contrast, the explicit thinking mode supports an individual’s creativity by changing existing mental models and building new ones [3]. There appears to be a tradeoff between the tendency to employ a previously learned heuristic mode and the ability to adopt an explicit mode [1,5]. The explicit mode might be an essential part of the creative process wherein an individual changes a conventional habit into a novel route of thinking [4].

Although studies in cognitive science [6] and evolutionary psychology [2] have explained the change from the heuristic mode to the explicit mode using environmental pressure, creativity studies [4,7] emphasize the role of an individual’s imagination in this change. A person could change a conventional representation by mentally forming alternative new representations. By using these alternative new representations, a person can exceed a conventional habit and enter a novel route of thinking and innovation [4,7].

Neuroscientific research has revealed valuable insights into brain activation related to creativity in the contexts of verbal and figurative insights, mental imagery, creative story generation, painting and melodies [8–15]. The most significant findings of these neuroscientific studies are the heteromodal cognitive regions of the frontal, temporal, parietal and limbic lobes [16]. Similarly, the executive network and the default mode network (DMN) appear to play important roles in creative thought [2,11,17,18]. The executive network most prominently includes the dorsolateral prefrontal cortex (DLPFC) and the anterior cingulate cortex (ACC). The default mode network (DMN) most prominently includes the medial prefrontal cortex (MPFC), posterior cingulate cortex (PCC), precuneus (PCU), temproparietal junction (TPJ) and inferior parietal lobes (IPL), including the angular (AG) and supramarginal gyrus (SMG). In addition, the role of the salience network may be important when an individual redirects a conventional habit to a new route. Although the insula is part of the salience network [19] and has been found to play essential role in emotional processing [20,21] its role in creativity is unclear.

Current studies emphasize that creativity involves not only capacity for explicit and heuristic thinking, but the capacity to shift dynamically between the two thinking modes according to the situation [22,23]. In order to uncover this dynamic process, it is important to organize the experiment, in which participants could engage in dynamic creative process. During consecutive idea generation, ideas’ originality has been shown to increase with time and the fluency to decrease [23,24]. This phenomenon is called the serial order effect and is one of the most consistent findings in creativity studies [23,24]. Yet, we know of no neuroimaging studies tackling the brain activity behind this phenomenon. The present study attempts to clarify what brain regions contribute to the serial order effect during divergent thinking task

To identify specific brain networks of the explicit mode, we administered participants a visual Alternative Uses Task (AUT) in which they were asked to generate as many alternative uses of a pictured everyday object as possible [25]. We administered a functional magnetic resonance imaging (fMRI) paradigm to examine which brain regions were activated when the participants mentally generated alternative uses for common household items.

We had three new aspects in our study, comparing previous creativity researches. At first, we focused serial order effect on idea generation. The serial order effect have not been used straight thru before by fMRI [23]. Second aspect was non-verbal tasks, i.e. our participants generate ideas mentally without verbalization. On the contrary several creativity studies subjects have been asked to produce and give verbal responses during the idea generation [9,25,26]. Finally, our participants decided by themselves, how many ideas and how long they were going to work with the idea generation task in the scanner. Following the past serial order research and divergent thinking [23] the only limitation was 15 minutes total time inside the scanner.

## Materials and Methods

### Participants

Twenty healthy, right-handed students from the Laurea University of Applied Sciences participated in this study. Four subjects were discarded due to excessive motion and other artifacts in the preprocessing stage of the data analysis. Thus, the final results are based on 16 subjects (4 males, mean age: 31.3 years, range: 19–49 years). The ethics committee of the Hospital District of Helsinki and Uusimaa approved the study. The subjects provided written informed consent before participating. Due to national legislation regulating the ethics committees, we are not allowed to distribute the original fMRI or behavioral data

### Stimuli and experimental conditions

**Fig 1:**
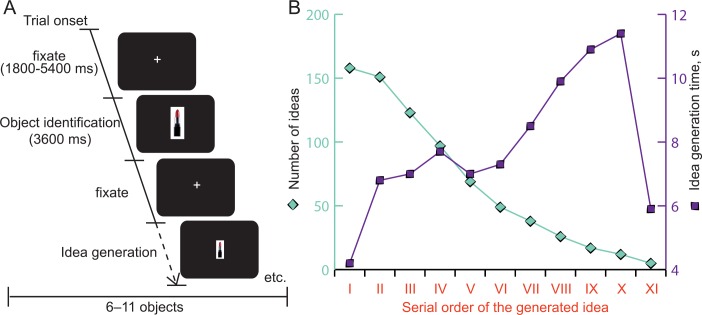
Stimulus design and behavioral results. **A)** The fMRI experiment lasted 15 minutes, during which time each participant was shown 6–11 household items depending on the participants’ individual performance. For each of the presented objects), the participants performed the task of first viewing the fixation cross for 1–3 TR (i.e. 1800–5400 ms). This was followed by the first stimulus presented as a picture of a common household item (duration 3600 ms). Then, the fixation cross was presented again for 1–3 TR. The participant was shown a smaller picture of the same household item and asked to mentally produce as many alternative uses as possible for the item. The participant indicated the initiation of this process, by pushing the fMRI-compatible button with the index finger. When the participant finished producing alternatives for the presented picture, he or she pushed a different button with the middle finger. Fluency across time during the fMRI-task is presented in **B).** The number of ideas (green) was highest at the beginning of the task and then dropped until the 11^th^ idea. However, the reaction time (blue) increased from the first to the tenth ideas but decreased again at the 11^th^ idea.

Participants performed the Alternative Uses Task [27] while being scanned in an fMRI scanner (Fig 1A). Each trial in the task started with a fixation screen for 1.8–5.4 s followed by the first stimulus. The first stimulus presented a picture of a common household item (duration 3.6 s). The task of the participants was to concentrate and view the item accurately (object identification phase). The original pictures shown were a tire, lipstick, coffee machine, plastic bottle, paper clip, blanket, wristwatch, tile, barrel, hammer, and wool sock. These items belong to categories that have been found to activate a large section of the ventral temporal cortex [28]. After the first stimulus, the participant was shown a smaller picture of the same item and asked to mentally produce as many alternative uses as possible for the object. Their task was to produce a lot of ideas. The participant indicated the initiation of this process by pushing the fMRI-compatible button with the index finger (idea generation phase). Thus, each response was time-stamped. When the participant was finished producing alternatives for the presented picture, a different button was pushed with the middle finger. This approach enabled the participants to decide for themselves how many alternative uses they created for each pictured object without time pressure.

We assumed that the object response in the temporal cortex maintained the object identity over changes in retinal size, indicating that the visual processing of these household items was object-identity-based rather than retinotopic [28]. In this way, we produced an experiment in which the difference between two stimuli (object identification vs. idea generation) had minimal contrast (i.e., the only difference was the size of the picture).

The subjects observed pictures through the mirror system. Each fMRI experiment lasted for 15 minutes. During this time each participant was shown 6–11 objects depending on the participants’ individual performance. Then, we counted the total number of ideas for each subject and used this total number for further analysis.

After scanning, the participants were asked to execute the same AUT using a paper and pencil. To keep the test situation in the scanner as purely idea generation and not a memory task, the participants did not previously know that they needed to execute the AUT after the fMRI experiment. The participants received the instruction: “On the following pages you will see different household items. Please produce as many alternative uses for each item as you can and write the ideas down”. Thus the subjects received no explicit request to recall their answers in the fMRI-AUT or to produce new ones.

### Data acquisition and analysis

We studied the fluency behavior by counting the total number of ideas produced during the fMRI test. Additionally, we studied the serial order effect by evaluating the response time required to produce the ideas. The limitations of the fMRI environment precluded recording of the generated ideas and, hence, evaluation of the ideas’ originality. Ideas’ originality in the serial order effect has been shown to increase with time and the fluency to decrease [23]. This fluency aspect of the serial order effect was readily assessable by counting the number of ideas and reaction times required to produce the ideas. The available behavioural data, thus, only allowed explicit evaluation of the fluency aspect of the serial order effect in AUT. To validate our fMRI-AUT approach, we counted the total ideas obtained from the paper and pencil test and calculated the correlation between the fMRI-AUT and the paper and pencil-AUT.

We acquired the MRI data on a 3 Tesla Siemens Magnetom Skyra (Siemens Medical Solutions, Erlangen, Germany). The imaging sequence parameters were: repetition time 1800 ms, flip angle 75 degrees, echo time 32 ms, field of view 22 cm, matrix size 64 × 64 (in-plane resolution 3.44 × 3.44 mm^2^), slice thickness 5 mm, and slice spacing 0. The imaging volume covered the whole brain with 27 contiguous oblique axial slices acquired in an interleaved fashion. Altogether, 500 volumes per functional run were acquired. The first volume of the recorded data was excluded from further analysis as a dummy. Additionally, three dummy scans were discarded by the scanner prior to recording the 500 volumes of the functional run.

Data analysis was performed in Matlab® v7.8.0347 (The MathWorks Inc., Natick, MA, USA) and was based on SPM8 (http://www.fil.ion.ucl.ac.uk/spm/software/spm8/; Welcome Department of Imaging Neuroscience London). All preprocessing was performed using the functional Data Processing Assistant (fDPA) toolbox developed by Yevhen Hlushchuk and Eerik Puuska based on the DPARSF toolbox [29]. In addition to DPARSF, the fDPA toolbox incorporates the ArtRepair toolbox [30].

The preprocessing steps included: realignment, normalization to the MNI space (the mean functional image was segmented and bias-corrected to improve coregistration to the anatomical space), and volume artifact removal (due to linear drift in the data we used the following thresholds in the ArtRepair toolbox: 5% signal change, rapid-movement threshold of 0.5 mm and z-threshold at 2.5). Datasets that required correction of more than 7% of the time-points were discarded from further analysis; subjects whose head movements during the recording exceeded 2 mm and 2 degrees were likewise discarded. After artifact removal, the data were smoothed at a full-width-at-half-maximum of 8 mm. The high-pass filter was set to the SPM8 default value of 128 s.

The preprocessed data subsequently underwent general linear model (GLM) analysis at the subject level. The model incorporated three predictors of interest: (i) “presentation”, (ii)”idea generation”, and (iii) “serial order”. The conditions were compared to each other to identify the brain regions that demonstrated relatively increased recruitment during the object identification and idea generation conditions (explicit > heuristic and heuristic > explicit). The ‘serial order’ was the linear parameter and encoded the serial order of creative representation a subject was working with for a given picture. Respective contrast images were generated for each subject and then entered into a second-level random effects analysis using a one-sample *t*-test. In the group-level statistical maps, the cluster-forming (voxel-level) threshold was set to p_uncorrected_ = 0.001 (quite commonly used in fMRI studies and recommended by recent studies on thresholding in fMRI) [31,32].

The threshold extent was set to 50 voxels and the clusters at *p* < 0.01 (FDR-corrected at the cluster level) were deemed significant.

## Results

### Behavioral results

The participants completed functional runs inside the fMRI scanner and afterwards executed the same task using a paper and pencil. The range of the number of ideas that participants produced for the given 11 objects during the fMRI task ranged from 22 to 148 (M = 71.06, SD = 32.88). Two participants generated ideas for 6 household items, two for 9 items, four for 10 items, and eight for 11 items. When completing the paper and pencil AUT outside of the scanner after the fMRI task, the range of the total number of ideas for the same household items ranged from 16 to 87 (M = 42.44, SD = 15.74). The number of button pushes inside the scanner correlated positively with the number of alternative uses written on paper (R = 0.71, R square = 0,509, p<0, 01). We found a clear serial order effect (Fig 1B). Thus the fluency was highest at the beginning of the task (M = 9.88, SD = 1.67) and then dropped at the 11^th^ idea (M = 1.25, SD = 0.5). However, the reaction time increased from the first (M = 4.2 sec, SD = 2.6 sec) to the tenth idea (M = 11.14 sec, SD = 4.66 sec) and unexpectedly decreased again at the 11^th^ idea (M = 5.9 sec, SD = 3.5 sec).

### FMRI results

**Fig 2:**
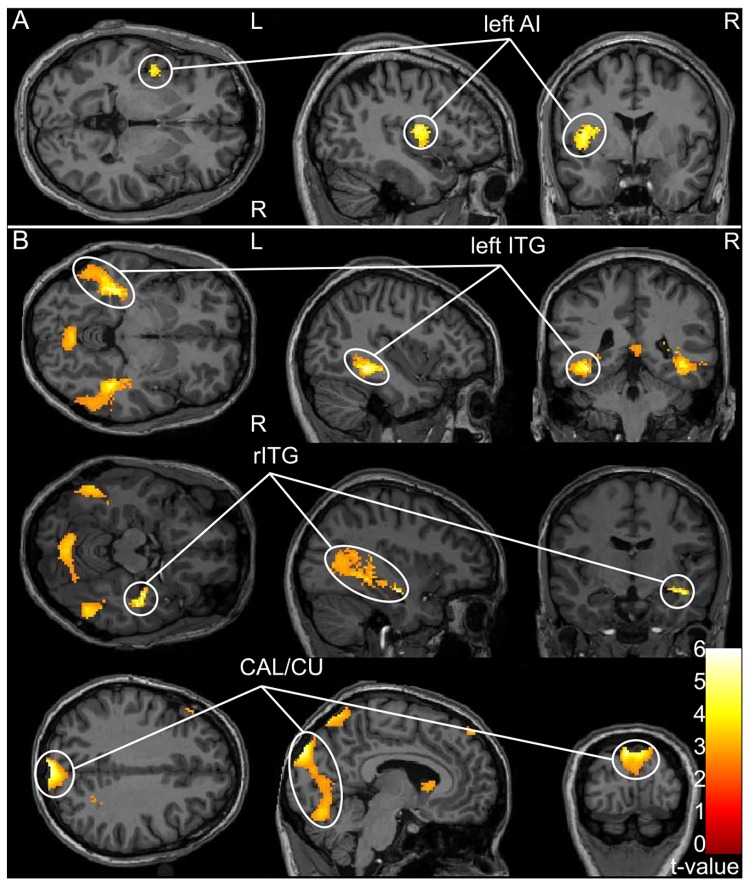
Brain regions showing significant differences based on comparisons between presentation and idea generation. **A)** Activations in the left anterior insula (lAI) region associated with idea generation relative to presentation. **B)** Activations in three clusters primarily located in the visual areas associated with presentation relative to idea generation (lITG—left inferior temporal gyrus; rITG–right inferior temporal gyrus; CAL/CU–anterior parts of visual areas on both dorsal and ventral sides of the calcarine sulcus). In A and B, the threshold at the voxel-level is p < 0.001 with an uncorrected cluster-size threshold set at 50 normalized voxels. All clusters surpassed the cluster-level threshold at p< 0.01 (FDR-corrected).

When the responses during the idea generation phase were contrasted to the presentation phase (Fig 2A, Table 1), the contrast revealed significant activation in the anterior left insula (-42, -4, -12) in Montreal Neurological Institute (MNI) space [33]. The opposite contrast in Fig 2B exposed three clusters in visual areas: Two clusters encompassed the left inferior temporal gyrus (ITG; -40, -42, -8) and right inferior temporal gyrus (36, -14, -16) and the third cluster encompassed anterior parts of visual areas on both dorsal and ventral sides of the calcarine sulcus (-6, -92, 34).

**Table 1:** Brain regions revealed by “idea generation > presentation” and “presentation > idea generation” contrasts.

	Anatomical region	MNI coordinates of global/local cluster's peak (x, y, z)	Cluster's size (in 2 x 2 x 2 mm^3^ voxels)	Peak-level, T-value	Cluster-level, FDR-corrected (q)
*Idea generation > presentation*					
	AI left	**-42, -4, 12**	**312**	**6.96**	**0.001**
		-34, 2, 12		5.66	
		-44, -2, 0		5.55	
*Presentation > idea generation*					
	ITG left	**-40, -42, -8**	**1309**	**8.45**	**0.000**
		-38, -34, -10		7.86	
		-32, -68, 8		7.03	
	CAL/CU	**-6, -92, 34**	**3437**	**8.05**	**0.000**
		2, -90, 34	** **	7.74	** **
		6, -74, 8	** **	6.87	** **
	ITG right	**36, -14, -16**	**1868**	**7.98**	**0.000**
		44, -18, -16		7.72	
		40, -38, -6	** **	6.98	** **

Significant clusters from the group level analysis (random effects, N = 16 subjects) are presented. The cluster-forming threshold was set at p < 0.001 (uncorrected); minimum cluster size 50 voxels. All clusters surpassed the cluster-level threshold at p< 0.01 (FDR-corrected). For clusters exceeding 200 voxels in size, up to two local submaxima 8 mm apart are also listed. Abbreviations: *AI–anterior insula*, *ITG–inferior temporal gyrus*, *CAL/CU–calcarine/cuneus*.

The GLM analysis included also a linear parameter (serial order regressor) that encoded the number of alternative ideas for a given picture applied to the estimated regressor coefficient maps. A conventional linear parameterization was used in which the first imagined idea was weighted by one, the second imagined use was weighted by 2, and so on. This linear parameterization model was used to detect regions in which the activity increased or decreased with serial order.

**Fig 3:**
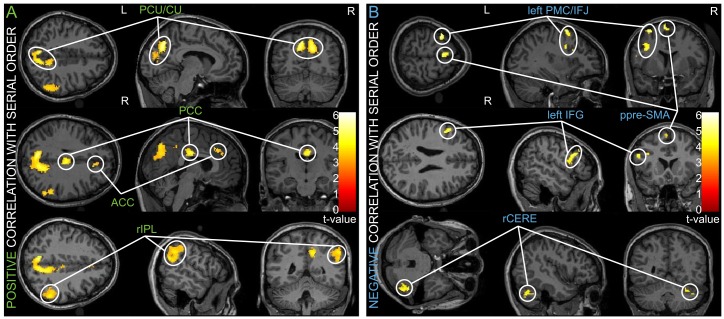
Brain regions correlating with the serial order. **A)** The amplitude of the BOLD signal positively correlated with the serial order regressor in four clusters (PCU–precuneus; CU–cuneus; PCC–posterior cingulate cortex; ACC–anterior cingulate cortex; rIPL—right inferior parietal lobe). **B)** Clusters that exhibited an opposing, negative correlation with the serial order regressor (ppre-SMA–posterior presupplementary motor area; left PMC/IFJ–left premotor cortex/inferior frontal junction; left IFG–left inferior frontal gyrus; rCERE–right cerebellum). The threshold at voxel-level was p < 0.001 uncorrected with the cluster-size threshold set at 50 normalized voxels. All clusters surpassed the cluster-level threshold at p < 0.01 (FDR-corrected).

The serial order in divergent thinking correlated (Fig 3A, Table 2) positively with the activation in the bilateral precuneus/cuneus (PCU/CU; -8, -66, 42). Their common behavior was supported by the underlying connectivity demonstrated for the dorsal precuneus and the ventral cuneus [34], PCC (4, - 20, 30) correlated with task difficulties [35], ACC (8, 30, 32) and the right inferior parietal lobule (rIPL; 56, -48, 38), including the supramarginal and angular gyrus.

**Table 2:** Brain areas where fMRI signal significantly correlated with the “serial order” predictor, positively or negatively.

	Anatomical region	MNI coordinates of global/local cluster's peak (x, y, z)	Cluster's size (in 2 x 2 x 2 mm^3^ voxels)	Peak-level, T-value	Cluster-level, FDR-corrected (q)
*"Serial order" positive*					
	PCU/CU	**-8, -66, 42**	**1725**	**7.24**	**0.001**
		-10, -68, 34		7.21	
		12, -62, 36		7.01	
	PCC	**4, -20, 30**	**189**	**5.71**	**0.001**
	IPL right	**56, -46, 30**	**597**	**5.06**	**0.001**
		56, -48, 38		4.97	
		56, -56, 26		4.59	
	ACC	**8, 30, 32**	**87**	**4.69**	**0.01**
*"Serial order" negative*					
	ppreSMA left	**-8, 6, 68**	**139**	**6.36**	**0.001**
	PMC/IFJ left	**-34, 4, 62**	**260**	**6.32**	**0.001**
		-36, 2, 34		5.24	
	IFG left	**-52, 16, 26**	**178**	**3.93**	**0.001**
	Cerebellum right	**44, -54, -34**	**282**	**3.93**	**0.001**

Significant clusters from the group level analysis (random effects, N = 16 subjects) are presented. The cluster-forming threshold was set at p < 0.001 (uncorrected); minimum cluster size 50 voxels. All clusters surpassed the cluster-level threshold at p< 0.01 (FDR-corrected). For clusters exceeding 200 voxels in size, up to two local submaxima 8 mm apart are also listed. Abbreviations: *PCU/CU–precuneus/cuneus*, *PCC- posterior cingulate cortex*, *IPL–inferior parietal lobule*, *ACC–anterior cingulate cortex*, *ppreSMA–posterior presupplementary motor area*, *PMC/IFJ–premotor cortex/inferior frontal junction*, *IFG–inferior frontal gyrus*.

The serial order correlated negatively (Fig 3B) in the posterior presupplementary motor area (ppreSMA; -8, 6, 68) in accordance with the functional connectivity segregation in the previous fMRI study [36], the left premotor cortex (left PMC; -34, 4, 62) with the major part of the cluster situated deep in the inferior precentral sulcus corresponding to the inferior frontal junction (IFJ) [37], the right cerebellum (44, -54, -34) and the left inferior frontal gyrus (IFG; -52, 16, 26).

## Discussion

The current study examined the hypothesis that idea generation was associated with the explicit thinking mode via the activation of neural processes related to the executive, default mode and salience networks. The study separated presentation from idea generation to uncover the neural substrate during idea generation. The study employed a novel paradigm in which the difference between object identification and idea generation had minimal contrast (i.e., the only difference was the size of the picture). This approach allowed us to separate and alternate between the heuristic thinking mode and explicit thinking mode. Previous creativity studies have emphasized the verbal production of ideas [11,23], whereas the present study allowed the participants to produce the ideas mentally without a verbal response. In addition, the study allowed the participants to decide freely, how long and how many ideas they like to produce without external time pressure or the demand to memorize their answers.

The findings of the study identify neural systems that play a role in idea generation and provide evidence that both the executive network and the DMN play roles in creative performance. Consistent with the hypothesized role of the executive network in idea generation, the participants showed increased activation in the ACC during this process. Similarly, consistent with the hypothesized role of the DMN in idea generation, the participants showed increased recruitment of the PCU/CU, PCC and IPL.

When contrasting the fMRI predictors for idea generation and presentation (explicit>heuristics), we observed activation in the left anterior insula. The opposite contrast showed significant activation in two clusters encompassing the left and right ITG.

During idea generation the serial order negatively correlated with the BOLD responses in the left ppreSMA, left PMC, right cerebellum and left IFG. According to Baddeley [38], the areas in premotor cortex, inferior frontal cortex and inferior parietal cortex have associations to the working memory–on the left side for verbal working memory and on the right side for visual working memory. In addition, working memory tasks have been consistently linked to widespread bilateral fronto-parietal networks including the IFG with Broca’s area, the PMC and pre-SMA [39,40]. Furthermore, the cerebellum is known to connect to the fronto-parietal cortices by mediating attentional processes [40]. Thus, the decreasing activation of the ppreSMA, cerebellum and IFG during idea generation suggests that idea generation without a verbal processing demand does not lead to increased load of the verbal working memory in the brain. On the contrary, their activity rather decrease reflecting lack of need for new object identification in consecutive idea generation events. The positive correlation of rIPL to the serial order instead could indicate the role of the visual working memory in the idea generation. The importance of this latter finding deserves more investigation.

Similarly, Hinds et al. [41] showed that greater activation in the SMA and lesser activation in the default-mode regions predicted superior vigilance. Whereas in vigilance tasks DMN activation indicates a “bad brain state” [see 41], in creative process it might be indicative of a “good brain state”. Thus, the current results imply that creative process may be multimodal and do not necessarily require resources from the brain’s verbal attentional and verbal working memory networks [42–44].

Behavioral results based on reaction times revealed that new ideas were easier to produce at the beginning compared to later in the process. These behavioral results confirm previous findings that cognitive and emotional pressures increase over time during idea generation [11,23].

The goal of the study was to find the correlation between serial order effect in divergent thinking and brain activity. It is possible, that participants idea generation strategies did change during this process as previous studies have found [45]. At the beginning they probably produced ideas based on memory, whereas later based on new associative combinations [22].

### The role of the executive networks in idea generation

The explicit mode may facilitate the change from the predefined interpretation of the object to a more novel interpretation by using the executive network. The executive network has been previously consistently linked to the inhibition of irrelevant information patterns [45] and the support of an individual when amplifying internal representations [2]. This network activates when predefined heuristic-based processes are not sufficient to deal with the current task [2].

Previously, the functions of the executive network have been attributed to the ACC and DLPFC [2,11]. Of these two areas, the current study only discovered activation in the ACC. Although the ACC has been previously assigned to the salience network [46,47], recent brain imaging articles on creative thinking, however, classify the ACC to the executive network [11,17,48]. The lack of significant DLPFC activation during idea generation was unexpected because the association between the prefrontal regions and creativity was consistently reported in neurophysiologically oriented studies [7,12,16,49]. Dietrich and Kanso [7] proposed that the association between the prefrontal regions and idea generation was related to the engagement of the working memory and attentional networks in the brain. Their argument was comprehensible because most previous creativity studies were based on either spoken or written verbal processes. In contrast, in the present study the task was to discover the counterfactual meaning of a conventional picture without the need of verbalizing. Thus, the involvement of the WM and other frontal cortical areas may not be necessary for a human to produce ideas without verbal elements. The importance of the prefrontal cortex in the different creativity contexts (verbal, non-verbal, tactual) deserves more investigation.

The role of the ACC as part of the executive network has been shown to be essential for processing novel or conflicting information from the environment [2] and maintaining task goals [50]. Thus, the executive network in general and the ACC in particular may enable the participants to redirect their conventional habit of thinking to a novel route.

### The role of the DMN in idea generation

The present study found an association between serial order in divergent thinking and activation in the PCU/CU, PCC and IPL including the AG and SMG, which are essential components of the DMN. We did not find any association between idea generation and MPFC activation.

Recruitment of the DMN is not typically linked with the explicit thinking mode. However, there is increasing evidence that the DMN may engage in a range of cognitive, affective and visceroceptive processes in addition to the resting state processes [11]. For example, the DMN is activated during memory retrieval, future planning, hypothesis generation and evaluation of the perspectives of others [51]. Studies by Limb and Braun [52] and Ellamil et al. [11] provided evidence for an association between creativity and the increased activation of the DMN. Moreover, previous studies showed that creative individuals were incapable of suppressing the activation of the DMN [53].

However, previous studies have also found that part of the DMN was deactivated during creative tasks. For example, negative associations have been reported between divergent thinking and activation in the PCU and TPJ as well as in the PCC and MCC [9]. One explanation for these contradictory results between the previous study and the current study may be that previous participants needed to vocalize [9] or write [26] their responses, whereas in our study the participants imagined their responses without verbalizing.

The evidence from the present study supported current theories that proposed a more general function for the DMN in processing internally generated cognitive and affective information rather than serving only as the resting state [11,51,54]. According to this theory, the DMN is in a readiness state and comprises tonically active regions of the brain that continuously gather information from the world around and within us [55]. The DMN begins to help other brain circuits when something unexpected or novel happens in the individual’s environment. When an individual monitors and integrates information about the world and within himself/herself, the DMN may facilitate the formation of emotional reactions that individuals monitor during information processing. This process may especially occur in the right IPL because it is located at the crossroads of areas specialized in processing many cognitive and affective functions that range from language processing to visual and number processes. Furthermore, the right IPL has been found critical for both conceptual metaphors and cross-modal abstractions and can function as a general convergence zone in the brain [56–58].

Previous studies have also produced new information concerning a more versatile role for the PCU [59] and PCC [35] as part of human cognition. In the current study, the PCU/CU cluster activation coordinates (-8, -66, 42) were almost the same as those reported in the Utevsky et al. [59] study. Their observation indicated that the PCU played a role not only in the DMN but also in cognition; this latter role was broader due to its engagement under a variety of processing states [59]. In the same vein, Leech et al. [35] proposed that the PCC was a hub within the brain that connected brain regions that supported complex behavior.

More generally, the PCC and PCU play roles in integrating information from the IPL and other brain regions by representing the relevant internally generated information [11,51]. As parts of the DMN, the IPL, PCU, and PCC have essential roles in idea generation by producing new combinations and mental models during the creative process.

### The role of the salience network in idea generation

A majority of past work has shown that the right anterior insula [46,47] and bilateral insula [21,47], as a core hub of the salience network, may facilitate interaction of the default and executive networks. Many recent brain imaging studies on creative thinking, however, have found the activation of the left insula during creative process as well [9,11,60,61]. Recent meta-analysis of fMRI studies on creativity has further exposed apparent left dominance for insula activation in non-verbal tasks [61]. Considering the non-verbal nature of the task in our experiment, the left anterior insula activation during idea generation in the current study is in line with previous studies.

The essential role of the insula in emotional processing implies that it may be important for creative thought by facilitating the ability of the central executive network and DMN to notice emotionally promising counterfactual elements in the environment and associations in the mind [21]. The role of the anterior insula as a core hub of the bilateral salience network [47,48] may be important when an individual redirects a conventional habit of thinking to a new route and our results lend further support to the claim that the lateral dominance of the anterior insula activation depends on the verbality/non-verbality of the task.

### Coactivations of the brain’s creative networks

The current study indicates that cognitive control is an essential part of serial order in divergent thinking. During this process the cognitive control supports the explicit thinking mode. However, idea generation was also associated with the recruitment of areas not typically associated with explicit thinking, such as the DMN and the salience network. Previously, the executive network and the DMN were proposed to act in opposition to one another such that the stimulus-independent DMN became deactivated when the stimulus-dependent executive network became activated and vice versa [11,18,62–64]. However, more recent creative studies have found evidence of coactivation in parts of the central executive network and the DMN, such as the ACC, PCC, PCU, and TPJ during insight problem solving [65,66] and the DLPFC, ACC, PCC, PCU and TPJ during a fluid analogy task [67]. Similar co-activation was found in the context of autobiographical planning [68], narrative speech comprehension [69], mind wandering [70] and continuous film viewing [71]. Thus, it appears that serial order in divergent thinking may allow for the combination of both the central executive network and the DMN. Our findings corroborate previously proposed notion that creative/divergent thinking involves interactions of the default, salience, and executive networks [11,48,52,60,72].

## Conclusions

The current study showed that regions of the executive, default mode—and salience networks were involved in the serial order in divergent thinking. Thus, the results indicate that an individual needs to suppress external stimuli and concentrate on the inner creative process during idea generation. This process may require a combination of elements that have little association and are isolated from the heuristic process. Our results indicate that the central executive network and the DMN together with salience network support focused and goal-oriented creative processes. The creative process is not “resting”; rather, it is a focused and goal-oriented process that uses inner cognitive and affective mental resources. Thus, the current results suggest that explicit thinking is an essential part of idea generation. The central executive network, DMN and salience networks may cooperate in this process.

In contrast to previous creativity studies, the current study found no association between frontal regions during idea generation, possibly because the task in the current study lacked enforcement of verbal processing during idea generation. Current approaches to mental representations [43,44] emphasize that they are multimodal in nature, which implies that the mental processing during idea generation does not have to be limited to a conceptual level. Eliasmith [73] proposes that when people are thinking about an object, they do not merely activate words that are associated with this object but seem to implicitly activate representations that are typical for the objects’ background. In this way, the human brain brings up emotional associations with the object and activates tactile, auditory, and visual representations of the object. The results of the current study support this approach of multimodal mental representation.

In addition, recent creativity studies [22,23] emphasize that creativity is neither heuristic nor explicit, but rather involves capacity to spontaneously shift between different kinds of thinking modes according to the demand of the context. In our serial order AUT experiment, our participants needed to produce alternative uses for everyday object.

Thus, imagining new mental possibilities and the willingness to change the conventional habit of thinking share the common process of cognitive control and the need for the DMN to construct new mental models, be it new options or new creative uses for an object.

## References

[1] Chrysikou EG, Thompson-Schill SL. Dissociable brain states linked to common and creative object use. Hum Brain Mapp. 2011;32: 665–675. 10.1002/hbm.21056 20533561 PMC3846690

[2] Geary D C. Origin of Mind Evolution of brain, cognition, and general intelligence. Washington: American PSychological Association; 2005.

[3] Luo J, Li W, Qiu J, Wei D, Liu Y, Zhang Q. Neural Basis of Scientific Innovation Induced by Heuristic Prototype. PLoS ONE. 2013;8: e49231 10.1371/journal.pone.0049231 23372641 PMC3555868

[4] Suomala J, Taatila V, Siltala R, Keskinen S. Chance Discovery as a First Step to Economic Innovation. Proceedings of the CogSci 2006, 28th annual conference of the Cognitive Science Society in cooperation with the 5th International Conference of the Cognitive Science: July 26–29, 2006, Vancouver, BC., Canada.(pp.2204-2209). Lawrence Erlbaum Ass.; 2006.

[5] Heilman KM, Nadeau SE, Beversdorf DO. Creative innovation: possible brain mechanisms. Neurocase. 2003;9: 369–379. 10.1076/neur.9.5.369.16553 14972752

[6] Thagard P. How Brains Make Mental Models In: Magnani L, Carnielli W, Pizzi C, editors. Model-Based Reasoning in Science and Technology. Springer Berlin Heidelberg; 2010 pp. 447–461. Available: http://link.springer.com/chapter/10.1007/978-3-642-15223-8_25

[7] Dietrich A, Kanso R. A review of EEG, ERP, and neuroimaging studies of creativity and insight. Psychol Bull. 2010;136: 822–848. 10.1037/a0019749 20804237

[8] Aziz-Zadeh L, Kaplan JT, Iacoboni M. “Aha!”: The neural correlates of verbal insight solutions. Hum Brain Mapp. 2009;30: 908–916. 10.1002/hbm.20554 18344174 PMC6870806

[9] Benedek M, Jauk E, Fink A, Koschutnig K, Reishofer G, Ebner F, et al To create or to recall? Neural mechanisms underlying the generation of creative new ideas. Neuroimage. 2014;88: 125–133. 10.1016/j.neuroimage.2013.11.021 24269573 PMC3991848

[10] Berkowitz AL, Ansari D. Expertise-related deactivation of the right temporoparietal junction during musical improvisation. NeuroImage. 2010;49: 712–719. 10.1016/j.neuroimage.2009.08.042 19715764

[11] Ellamil M, Dobson C, Beeman M, Christoff K. Evaluative and generative modes of thought during the creative process. Neuroimage. 2012;59: 1783–1794. 10.1016/j.neuroimage.2011.08.008 21854855

[12] Fink A, Grabner RH, Benedek M, Reishofer G, Hauswirth V, Fally M, et al The creative brain: investigation of brain activity during creative problem solving by means of EEG and FMRI. Hum Brain Mapp. 2009;30: 734–748. 10.1002/hbm.20538 18266217 PMC6871103

[13] Goel V, Vartanian O. Dissociating the roles of right ventral lateral and dorsal lateral prefrontal cortex in generation and maintenance of hypotheses in set-shift problems. Cereb Cortex. 2005;15: 1170–1177. 10.1093/cercor/bhh217 15590912

[14] Howard-Jones PA, Blakemore S-J, Samuel EA, Summers IR, Claxton G. Semantic divergence and creative story generation: an fMRI investigation. Brain Res Cogn Brain Res. 2005;25: 240–250. 10.1016/j.cogbrainres.2005.05.013 15993573

[15] Jung-Beeman M, Bowden EM, Haberman J, Frymiare JL, Arambel-Liu S, Greenblatt R, et al Neural Activity When People Solve Verbal Problems with Insight. PLoS Biol. 2004;2: e97 10.1371/journal.pbio.0020097 15094802 PMC387268

[16] Arden R, Chavez RS, Grazioplene R, Jung RE. Neuroimaging creativity: a psychometric view. Behav Brain Res. 2010;214: 143–156. 10.1016/j.bbr.2010.05.015 20488210

[17] Beaty RE, Benedek M, Wilkins RW, Jauk E, Fink A, Silvia PJ, et al Creativity and the default network: A functional connectivity analysis of the creative brain at rest. Neuropsychologia. 2014;64: 92–98. 10.1016/j.neuropsychologia.2014.09.019 25245940 PMC4410786

[18] Raichle ME, Snyder AZ. A default mode of brain function: a brief history of an evolving idea. Neuroimage. 2007;37: 1083–1090; discussion 1097–1099. 10.1016/j.neuroimage.2007.02.041 17719799

[19] Seeley WW, Menon V, Schatzberg AF, Keller J, Glover GH, Kenna H, et al Dissociable Intrinsic Connectivity Networks for Salience Processing and Executive Control. J Neurosci. 2007;27: 2349–2356. 10.1523/JNEUROSCI.5587-06.2007 17329432 PMC2680293

[20] Bechara A, Damasio AR. The Somatic Marker Hypothesis: A Neural Theory of Economic Decision. Games and Economic Behavior. 2005;52: 336–372.

[21] Cauda F, D’Agata F, Sacco K, Duca S, Geminiani G, Vercelli A. Functional connectivity of the insula in the resting brain. Neuroimage. 2011;55: 8–23. 10.1016/j.neuroimage.2010.11.049 21111053

[22] Gabora L. Revenge of the “Neurds”: Characterizing Creative Thought in Terms of the Structure and Dynamics of Memory. Creativity Research Journal. 2010;22: 1–13. 10.1080/10400410903579494

[23] Beaty RE, Silvia PJ. Why do ideas get more creative across time? An executive interpretation of the serial order effect in divergent thinking tasks. Psychology of Aesthetics, Creativity, and the Arts. 2012;6: 309–319. 10.1037/a0029171

[24] Silvia PJ, Beaty RE. Making creative metaphors: The importance of fluid intelligence for creative thought. Intelligence. 2012;40: 343–351. 10.1016/j.intell.2012.02.005

[25] Fink A, Grabner RH, Gebauer D, Reishofer G, Koschutnig K, Ebner F. Enhancing creativity by means of cognitive stimulation: evidence from an fMRI study. Neuroimage. 2010;52: 1687–1695. 10.1016/j.neuroimage.2010.05.072 20561898

[26] Fink A, Koschutnig K, Benedek M, Reishofer G, Ischebeck A, Weiss EM, et al Stimulating creativity via the exposure to other people’s ideas. Hum Brain Mapp. 2012;33: 2603–2610. 10.1002/hbm.21387 23074077 PMC6870350

[27] Guilford JP. The nature of human intelligence McGraw-Hill; 1967.

[28] Konkle T, Oliva A. A Real-World Size Organization of Object Responses in Occipitotemporal Cortex. Neuron. 2012;74: 1114–1124. 10.1016/j.neuron.2012.04.036 22726840 PMC3391318

[29] Chao-Gan Y, Yu-Feng Z. DPARSF: A MATLAB Toolbox for “Pipeline” Data Analysis of Resting-State fMRI. Front Syst Neurosci. 2010;4 10.3389/fnsys.2010.00013 PMC2889691 20577591

[30] Mazaika PK, Hoeft F, Glover GH, Reiss AL. Methods and Software for fMRI Analysis of Clinical Subjects. NeuroImage. 2009;47, Supplement 1: S58 10.1016/S1053-8119(09)70238-1

[31] Eklund A, Nichols T, Knutsson H. Can parametric statistical methods be trusted for fMRI based group studies? arXiv:151101863 [math, stat]. 2015; Available: http://arxiv.org/abs/1511.01863

[32] Woo C-W, Krishnan A, Wager TD. Cluster-extent based thresholding in fMRI analyses: Pitfalls and recommendations. Neuroimage. 2014;91: 412–419. 10.1016/j.neuroimage.2013.12.058 24412399 PMC4214144

[33] Evans AC, Collins DL, Mills SR, Brown ED, Kelly RL, Peters TM. 3D statistical neuroanatomical models from 305 MRI volumes. Nuclear Science Symposium and Medical Imaging Conference, 1993, 1993 IEEE Conference Record. 1993. pp. 1813–1817 vol.3. doi: 10.1109/NSSMIC.1993.373602

[34] Zhang S, Li CR. Functional connectivity mapping of the human precuneus by resting state fMRI. NeuroImage. 2012;59: 3548–3562. 10.1016/j.neuroimage.2011.11.023 22116037 PMC3288461

[35] Leech R, Kamourieh S, Beckmann CF, Sharp DJ. Fractionating the Default Mode Network: Distinct Contributions of the Ventral and Dorsal Posterior Cingulate Cortex to Cognitive Control. Journal of Neuroscience. 2011;31: 3217–3224. 10.1523/JNEUROSCI.5626-10.2011 21368033 PMC6623935

[36] Zhang S, Ide JS, Li C -s. R. Resting-State Functional Connectivity of the Medial Superior Frontal Cortex. Cerebral Cortex. 2012;22: 99–111. 10.1093/cercor/bhr088 21572088 PMC3236794

[37] Derrfuss J, Vogt VL, Fiebach CJ, von Cramon DY, Tittgemeyer M. Functional organization of the left inferior precentral sulcus: Dissociating the inferior frontal eye field and the inferior frontal junction. NeuroImage. 2012;59: 3829–3837. 10.1016/j.neuroimage.2011.11.051 22155041

[38] Baddeley A. Working memory: looking back and looking forward. Nat Rev Neurosci. 2003;4: 829–839. 10.1038/nrn1201 14523382

[39] Rottschy C, Langner R, Dogan I, Reetz K, Laird AR, Schulz JB, et al Modelling neural correlates of working memory: A coordinate-based meta-analysis. NeuroImage. 2012;60: 830–846. 10.1016/j.neuroimage.2011.11.050 22178808 PMC3288533

[40] Schlegel A, Kohler PJ, Fogelson SV, Alexander P, Konuthula D, Tse PU. Network structure and dynamics of the mental workspace. Proceedings of the National Academy of Sciences. 2013;110: 16277–16282. 10.1073/pnas.1311149110 PMC3791746 24043842

[41] Hinds O, Thompson TW, Ghosh S, Yoo JJ, Whitfield-Gabrieli S, Triantafyllou C, et al Roles of default-mode network and supplementary motor area in human vigilance performance: evidence from real-time fMRI. Journal of Neurophysiology. 2013;109: 1250–1258. 10.1152/jn.00533.2011 23236006

[42] Barsalou LW. Simulation, situated conceptualization, and prediction. Philosophical Transactions of the Royal Society B: Biological Sciences. 2009;364: 1281–1289. 10.1098/rstb.2008.0319 PMC2666716 19528009

[43] Eliasmith C, Stewart TC, Choo X, Bekolay T, DeWolf T, Tang Y, et al A Large-Scale Model of the Functioning Brain. Science. 2012;338: 1202–1205. 10.1126/science.1225266 23197532

[44] Thagard P, Stewart TC. The AHA! Experience: Creativity Through Emergent Binding in Neural Networks. Cognitive Science. 2011;35: 1–33. 10.1111/j.1551-6709.2010.01142.x 21428991

[45] Gilhooly KJ, Fioratou E, Anthony SH, Wynn V. Divergent thinking: strategies and executive involvement in generating novel uses for familiar objects. Br J Psychol. 2007;98: 611–625. 10.1348/096317907X173421 17535464

[46] Menon V, Uddin LQ. Saliency, switching, attention and control: a network model of insula function. Brain Structure and Function. 2010;214: 655–667. 10.1007/s00429-010-0262-0 20512370 PMC2899886

[47] Uddin LQ. Salience processing and insular cortical function and dysfunction. Nature Reviews Neuroscience. 2014;16: 55–61. 10.1038/nrn3857 25406711

[48] Beaty RE, Benedek M, Silvia PJ, Schacter DL. Creative Cognition and Brain Network Dynamics. Trends in Cognitive Sciences. 2016;20: 87–95. 10.1016/j.tics.2015.10.004 26553223 PMC4724474

[49] Chávez-Eakle RA, Graff-Guerrero A, García-Reyna J-C, Vaugier V, Cruz-Fuentes C. Cerebral blood flow associated with creative performance: A comparative study. NeuroImage. 2007;38: 519–528. 10.1016/j.neuroimage.2007.07.059 17884587

[50] Dosenbach NUF, Fair DA, Cohen AL, Schlaggar BL, Petersen SE. A dual-networks architecture of top-down control. Trends Cogn Sci (Regul Ed). 2008;12: 99–105. 10.1016/j.tics.2008.01.001 18262825 PMC3632449

[51] Buckner RL, Andrews-Hanna JR, Schacter DL. The Brain’s Default Network. Annals of the New York Academy of Sciences. 2008;1124: 1–38. 10.1196/annals.1440.011 18400922

[52] Limb CJ, Braun AR. Neural Substrates of Spontaneous Musical Performance: An fMRI Study of Jazz Improvisation. PLoS ONE. 2008;3: 1–9. 10.1371/journal.pone.0001679 PMC2244806 18301756

[53] Takeuchi H, Taki Y, Hashizume H, Sassa Y, Nagase T, Nouchi R, et al The association between resting functional connectivity and creativity. Cereb Cortex. 2012;22: 2921–2929. 10.1093/cercor/bhr371 22235031

[54] Bar M. The proactive brain: memory for predictions. Philosophical Transactions of the Royal Society of London B: Biological Sciences. 2009;364: 1235–1243. 10.1098/rstb.2008.0310 19528004 PMC2666710

[55] Cavanna AE, Trimble MR. The precuneus: a review of its functional anatomy and behavioural correlates. Brain. 2006;129: 564–583. 10.1093/brain/awl004 16399806

[56] Damasio AR. Time-locked multiregional retroactivation: a systems-level proposal for the neural substrates of recall and recognition. Cognition. 1989;33: 25–62. 2691184 10.1016/0010-0277(89)90005-x

[57] Ramachandran VS, Hubbard EM. The Phenomenology of Synaesthesia. Journal of Consciousness Studies. 2003;10: 49–57.

[58] Simmons WK, Barsalou LW. The Similarity-in-Topography Principle: Reconciling Theories of Conceptual Deficits. Cognitive Neuropsychology. 2003;20: 451–486. 10.1080/02643290342000032 20957580

[59] Utevsky AV, Smith DV, Huettel SA. Precuneus Is a Functional Core of the Default-Mode Network. Journal of Neuroscience. 2014;34: 932–940. 10.1523/JNEUROSCI.4227-13.2014 24431451 PMC3891968

[60] Beaty RE, Benedek M, Barry Kaufman S, Silvia PJ. Default and Executive Network Coupling Supports Creative Idea Production. Scientific Reports. 2015;5: 10964 10.1038/srep10964 26084037 PMC4472024

[61] Gonen-Yaacovi G, de Souza LC, Levy R, Urbanski M, Josse G, Volle E. Rostral and caudal prefrontal contribution to creativity: a meta-analysis of functional imaging data. Front Hum Neurosci. 2013;7: 465 10.3389/fnhum.2013.00465 23966927 PMC3743130

[62] Fox MD, Snyder AZ, Vincent JL, Corbetta M, Essen DCV, Raichle ME. The human brain is intrinsically organized into dynamic, anticorrelated functional networks. PNAS. 2005;102: 9673–9678. 10.1073/pnas.0504136102 15976020 PMC1157105

[63] Greicius MD, Krasnow B, Reiss AL, Menon V. Functional connectivity in the resting brain: a network analysis of the default mode hypothesis. Proc Natl Acad Sci USA. 2003;100: 253–258. 10.1073/pnas.0135058100 12506194 PMC140943

[64] Weissman DH, Roberts KC, Visscher KM, Woldorff MG. The neural bases of momentary lapses in attention. Nat Neurosci. 2006;9: 971–978. 10.1038/nn1727 16767087

[65] Kounios J, Fleck JI, Green DL, Payne L, Stevenson JL, Bowden EM, et al The origins of insight in resting-state brain activity. Neuropsychologia. 2008;46: 281–291. 10.1016/j.neuropsychologia.2007.07.013 17765273 PMC2293274

[66] Subramaniam K, Kounios J, Parrish TB, Jung-Beeman M. A brain mechanism for facilitation of insight by positive affect. J Cogn Neurosci. 2009;21: 415–432. 10.1162/jocn.2009.21057 18578603

[67] Geake JG, Hansen PC. Neural correlates of intelligence as revealed by fMRI of fluid analogies. NeuroImage. 2005;26: 555–564. 10.1016/j.neuroimage.2005.01.035 15907312

[68] Spreng RN, Stevens WD, Chamberlain JP, Gilmore AW, Schacter DL. Default network activity, coupled with the frontoparietal control network, supports goal-directed cognition. NeuroImage. 2010;53: 303–317. 10.1016/j.neuroimage.2010.06.016 20600998 PMC2914129

[69] Wilson SM, Molnar-Szakacs I, Iacoboni M. Beyond superior temporal cortex: intersubject correlations in narrative speech comprehension. Cereb Cortex. 2008;18: 230–242. 10.1093/cercor/bhm049 17504783

[70] Christoff K, Gordon AM, Smallwood J, Smith R, Schooler JW. Experience sampling during fMRI reveals default network and executive system contributions to mind wandering. Proceedings of the National Academy of Sciences. 2009;106: 8719–8724. 10.1073/pnas.0900234106 PMC2689035 19433790

[71] Golland Y, Bentin S, Gelbard H, Benjamini Y, Heller R, Nir Y, et al Extrinsic and intrinsic systems in the posterior cortex of the human brain revealed during natural sensory stimulation. Cereb Cortex. 2007;17: 766–777. 10.1093/cercor/bhk030 16699080

[72] Jung RE, Mead BS, Carrasco J, Flores RA. The structure of creative cognition in the human brain. Front Hum Neurosci. 2013;7: 330 10.3389/fnhum.2013.00330 23847503 PMC3703539

[73] Eliasmith C. How to Build a Brain: A Neural Architecture for Biological Cognition 1 edition Oxford: Oxford University Press; 2013.

